# Membrane-based inverse-transition purification facilitates a rapid isolation of various spider-silk elastin-like polypeptide fusion proteins from extracts of transgenic tobacco

**DOI:** 10.1007/s11248-024-00375-z

**Published:** 2024-04-04

**Authors:** H. M. Gruchow, P. Opdensteinen, J. F. Buyel

**Affiliations:** 1 Institute for Molecular Biotechnology, RWTH Aachen University, Worringerweg 1, 52074 Aachen, Germany; 2 Institute of Bioprocess Science and Engineering (IBSE), Department of Biotechnology (DBT), University of Natural Resources and Life Sciences (BOKU), Muthgasse 18, 1190 Vienna, Austria

**Keywords:** Downstream processing, Plant molecular farming, Process optimization, Spider silk proteins, Surface plasmon resonance spectroscopy, Ultrafiltration/diafiltration

## Abstract

Plants can produce complex pharmaceutical and technical proteins. Spider silk proteins are one example of the latter and can be used, for example, as compounds for high-performance textiles or wound dressings. If genetically fused to elastin-like polypeptides (ELPs), the silk proteins can be reversibly precipitated from clarified plant extracts at moderate temperatures of ~ 30 °C together with salt concentrations > 1.5 M, which simplifies purification and thus reduces costs. However, the technologies developed around this mechanism rely on a repeated cycling between soluble and aggregated state to remove plant host cell impurities, which increase process time and buffer consumption. Additionally, ELPs are difficult to detect using conventional staining methods, which hinders the analysis of unit operation performance and process development. Here, we have first developed a surface plasmon resonance (SPR) spectroscopy-based assay to quantity ELP fusion proteins. Then we tested different filters to prepare clarified plant extract with > 50% recovery of spider silk ELP fusion proteins. Finally, we established a membrane-based purification method that does not require cycling between soluble and aggregated ELP state but operates similar to an ultrafiltration/diafiltration device. Using a data-driven design of experiments (DoE) approach to characterize the system of reversible ELP precipitation we found that membranes with pore sizes up to 1.2 µm and concentrations of 2–3 M sodium chloride facilitate step a recovery close to 100% and purities of > 90%. The system can thus be useful for the purification of ELP-tagged proteins produced in plants and other hosts.

**Supplementary Information:**

The online version contains supplementary material available at 10.1007/s11248-024-00375-z.

## Introduction

Plants can have several advantages for the production of biopharmaceutical proteins such as low upstream production costs of ~ 50 € kg^−1^ wet biomass due to simple cultivation (Ridgley et al. 2023) and an inherent safety because they do not support the replication of human pathogens (Schillberg et al. 2019; Donini and Marusic 2019; Moustafa et al. 2016; Tschofen et al. 2016; Buyel 2023). However, the recovery of recombinant proteins from plant extracts can be challenging due to large quantities of particles and host cell proteins that are typically released during biomass homogenization (Wilken and Nikolov 2012; Buyel 2015). Various methods have been develop in the last years to address this issue, including flocculation or blanching to simplify clarification and purification respectively (Buyel et al. 2014; Buyel and Fischer 2014a).

Apart from process modifications, purification challenges can be circumvented by genetically engineering a product. For example, the product can be extended with short stretches of amino acids, so called tags, that simplify product capture (Costa et al. 2014; Pina et al. 2014; Young et al. 2012). One of the most commonly used tags facilitating affinity chromatographic purification is a stretch of about six histidine residues typically located at the N-terminus or C-terminus of a product. This tag enables selective binding of a product to immobilized divalent metal ions (Debeljak et al. 2006). However, the histidine tag may be inefficient for purification for example if chelating agents are present (Gengenbach et al. 2018). Also, the tag may be cleaved of in dependence of the plant cultivation conditions such as growth temperatures > 40 °C (Knödler et al. 2019) or the corresponding chromatography resin can be too costly for the bulk production of technical proteins. An example of the latter are spider silk proteins that can be used as high-performance textile fibers (Belbéoch et al. 2021).

Alternatively, elastin-like polypeptide (ELP) based purification tags offer a straightforward and simple chromatography-free separation of product and host cell proteins (HCPs). Also, ELPs can be effectively produced in plants and plant cells (Floss et al. 2010) and the tag can be cleaved-off after purification (Lan et al. 2011) so that authentic product can be recovered. ELPs comprise 30‒100 repeats of a VPGXG motif, where X is any amino acid except proline (Urry 1988). This sequence mediates a reversible aggregation of ELPs as well as ELP-fusion proteins (Christensen et al. 2013) at a so called transition temperature (*T*_*t*_), which is ≥ 30 °C in the presence of ≥ 1.0 M salt. The *T*_*t*_ can be reduced by increasing the salt concentration as well as by increasing number of VPGXG repeats in the ELP tag (Conley et al. 2009) as well as by increasing the hydrophobicity of the guest residue X in the ELP motif (Urry et al. 1992; Miao et al. 2003). Accordingly, the method is compatible with heat-labile fusion proteins (Bischof and He 2005). The aggregates formed by ELP fusion proteins are in the micrometer range (Miao et al. 2003) so that > 95% of soluble HCPs can be removed with the supernatant after centrifugation (Meyer and Chilkoti 1999) or in the flow-through of a membrane filtration step (Phan and Conrad 2011). The method is termed ‘inverse transition cycling’ (ITC) and it has been used successfully to purify spider silk proteins (Weichert et al. 2014), hemagglutinin (Phan et al. 2014) and lectins (Tian and Sun 2011).

**Fig. 1:**
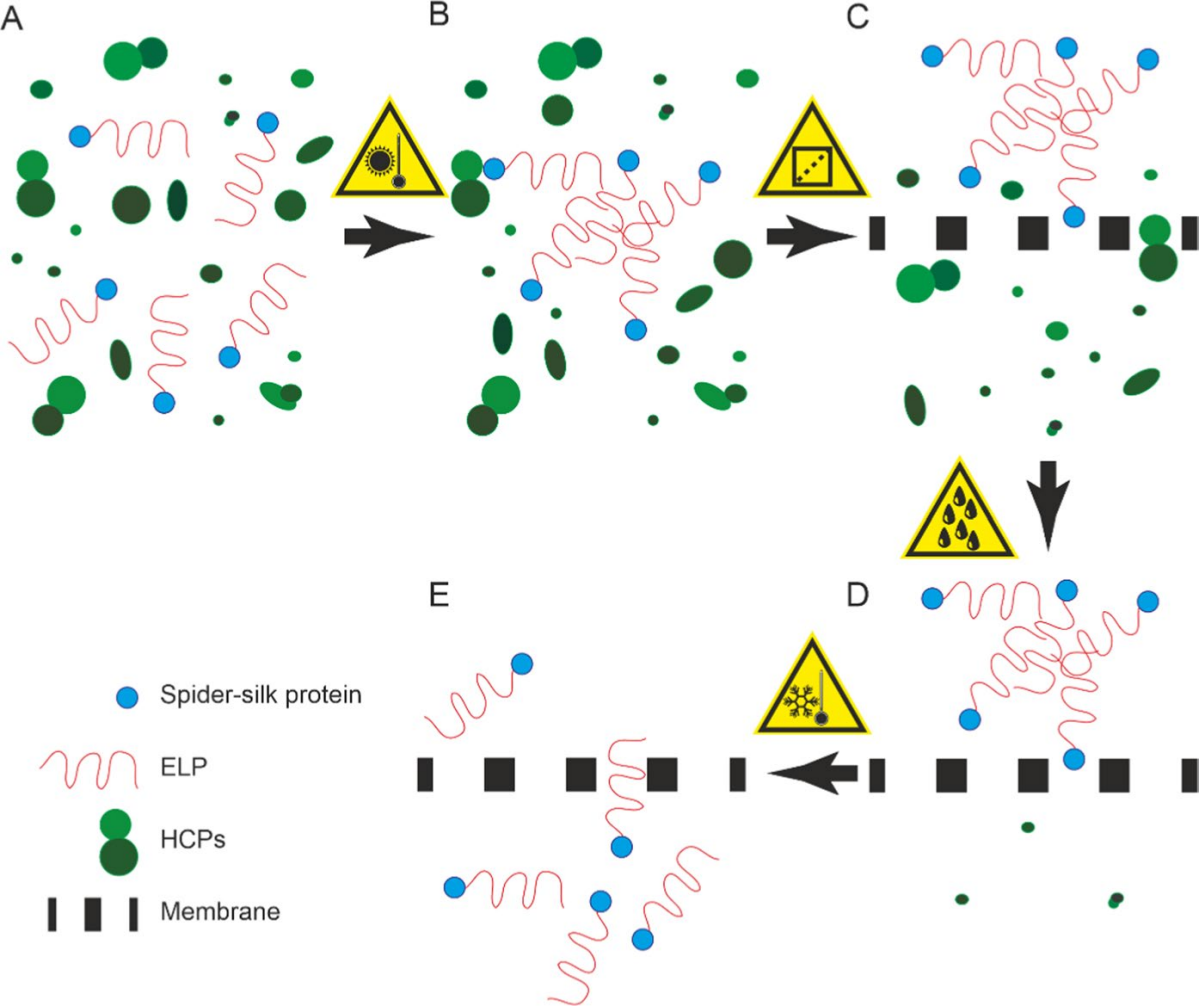
Schematic representation of the membrane-based inverse transition purification (mITP) process. Starting with a clarified plant homogenate (**A**), the temperature is increased in the presence of salt to trigger the precipitation of ELP-fusion proteins (**B**, here: fused to spider-silk proteins). The suspension is then applied to a membrane of suitable pore size (e.g. 0.2–2.0 µm), for example in an ultrafiltration/diafiltration device, so that the precipitate is retained whereas the bulk homogenate passes into the flowthrough (**C**). Next, the membrane is flushed with a hot, salt-rich buffer to remove residual impurities whereas the ELP-fusion proteins remain in a precipitated state (**D**). Lastly, a cold buffer (without salt) or plain water is used to re-dissolve the ELP precipitate and to elute the product from the membrane (**E**)

**Fig. 2:**
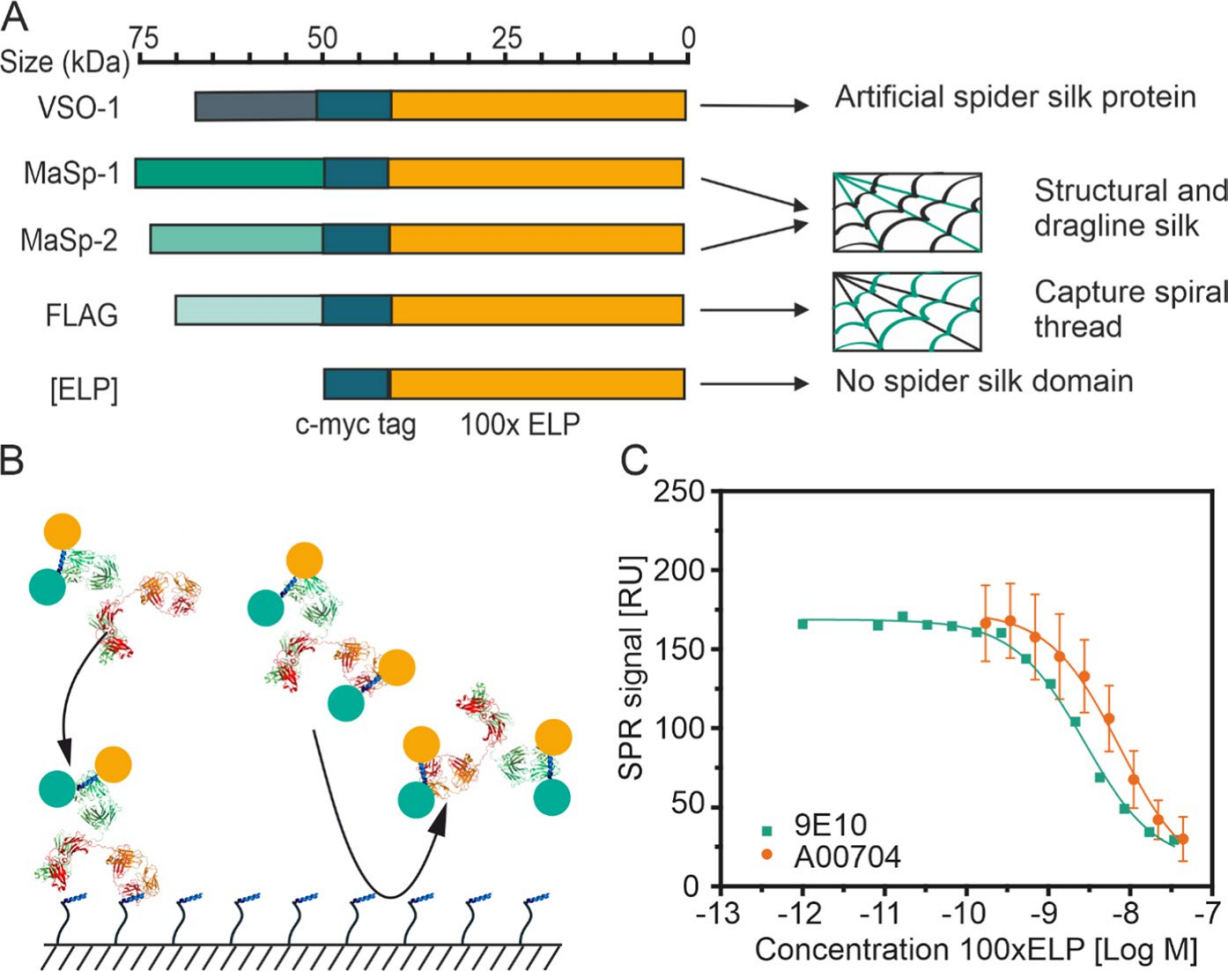
Spider silk elastin-like polypeptides (ELP) fusion proteins and their quantification of with a surface plasmon resonance (SPR) spectroscopy competitive binding assay. **A** Schematic representation of the five fusion proteins used in this study. The c-myc part of the fusion protein is shown in blue, whereas the spider-silk domain is colored in green and the ELP part is orange. **B** Competition assay principle. Anti-c-myc antibody (red) pre-incubated with c-myc-tagged ELP fusion protein (domain color code as in A) containing sample or standard is brought in contact with a surface decorated with peptides containing a c-myc epitope or variant thereof. Only antibodies with at least one unoccupied valency can bind to the surface resulting in a response signal. **C** Response resulting from antibody (green—9E10, n = 1; orange—A00704, n = 3) binding to a surface decorated with peptide 3 in dependence of the concentration of ELP standard the antibody was pre-incubated with. Data were fitted to a site competition model (Eq. 1) to derive inflection points

However, both centrifuge (cITC) and membrane-based (mITC) methods are currently operated in a discontinuous mode, requiring several aggregation-disaggregation cycles to achieve high product purity and have limited scalability. We therefore set out to investigate if ITC can be adapted to ultrafiltration/diafiltration and the corresponding good manufacturing practice-compliant equipment, which allows a simple scale-up and a continuous operation with only a single aggregation step because residual HCPs can be separated from the product in diafiltration operation mode (Fig. 1). We termed this approach ‘membrane-based inverse transition purification’ (mITP) to discriminate it from the previous methods that require cyclic processing of feeds. Five spider silk-ELP fusion proteins (Fig. 2A) were used to develop the method and to demonstrate its transferability.

## Materials and methods

### Plant material and cultivation

Transgenic *N. tabacum* VFlag-100×ELP line 5-6/2 (Flag, UniProtKB O44359), VMaSp1-100×ELP line 24-8/1 (MaSp1, UniProtKB Q8WSW4), VMaSp2-100×ELP line 56-3/1 (MaSp2, UniProtKB P46804), VSO1-100×ELP line 15-2/3 (VSO1, UniProtKB P19837) and 100xELP line 16 (100×ELP) (Scheller et al. 2001; Heppner et al. 2016; Hauptmann et al. 2015) were a kind donation of Dr. Udo Conrad of the Leibnitz Institute of Plant Genetics and Crop Plant Research (Gatersleben, Germany). Seeds were germinated in soil. The total cultivation time in a greenhouse (50°47′07.1′′N 6°03′00.5′′E) 50 days with 25/22 °C day/night temperature and 70% relative humidity using a 0.1% (m/v) solution of Ferty 2 Mega (Kammlott GmbH, Germany) for irrigation. Leaves were stored in plastic bags at − 20 °C after harvest until protein extraction.

### Protein structure, extraction and clarification

ELP fusion proteins FlagELP (61.6 kDa), MaSp1ELP (70.4 kDa), MaSp2ELP (74.6 kDa), VSO1ELP (58.8 kDa) and 100×ELP (45.6 kDa) consisted of an N-terminal spider silk protein part, a central c-myc tag (UniProtKB P01106) and 100 repetitions of the ELP motive VPGXG at the C-terminus (Phan and Conrad 2011) (Fig. 2A). A combination of signal peptide (N-terminus, UniProtKB P05190) and KDEL tag (C-terminus) was used to retain the protein in the endoplasmic reticulum.

Total soluble protein (TSP) was extracted from 0.15 to 2.00 kg of leaves of transgenic plants in blade-based homogenizers using 3 L extraction buffer (50 mM sodium phosphate, 500 mM sodium chloride, 10 mM sodium bisulfite, pH 8.0) per kilogram wet biomass as previously described (Buyel and Fischer 2014b). A sequence of bag (~ 1 µm pore size), depth (0.3–10.0 µm pore size) and sterile filters (0.2 µm pore size) was used for clarification as recently reported (Buyel and Fischer 2014b). Depth filtration was carried out with a linear flow rate of 0.15 m h^−1^ (2.5 L m^−2^ min^−1^) using different filters (Table S1) of 60 mm diameter (~ 0.003 m^2^). Alternatively, filtration steps were replaced by centrifuging samples twice at 16,000× *g* for 20 min at 4 °C. The supernatant was used for further testing.

### Membrane-based inverse transition purification screening

Clarified plant extract was used for mITP and the process parameters aggregation temperature, salt concentration during aggregation and wash as well as membrane pore size were optimized in terms of product purity and recovery using a 70-run (including 10 replicates) d-optimal response surface design with quadratic base model (Table S2). Design Expert v11 was used to set up and evaluate the experimental design. For each run, the conductivity was adjusted to the required value by adding sodium chloride to a 10-mL aliquot of extract, which was heated to the temperature defined in the experimental design using a water bath (E300, Lauda, Lauda-Königshofen, Germany). Then, 3 mL of sample were passed through 0.62 × 10^–3^ m^2^ membranes (Minisart, Sartorius Stedim, Göttingen, Germany) with pore sizes as required for the experiment (Table S2) using 5-mL single-use syringes (B.Braun, Melsungen, Germany). Residual liquid in the 0.15 mL filter dead volume was recovered by an injection of air followed by a 6-mL wash with the appropriate, pre-warmed solution. Proteins were eluted from the membranes in two fractions, first by passing 6 mL of ice-cold buffer (15 mM phosphate buffer pH 7.5) through the membrane followed by 6 mL of ice-cold water.

### Protein analysis and quantitation

The TSP concentration was determined using a microtiter version of the method of Bradford as described before (Buyel and Fischer 2014a) and TSP composition was analyzed by lithium dodecyl sulfate polyacrylamide gel electrophoresis (LDS-PAGE) with 4–12% (m/v) continuous Bis–Tris gradient gels followed by Coomassie-staining or silver-staining according to the manufacturers information (ThermoFischer, Carlsbad, USA).

Alternatively, gels were used for western-blot analysis with a commercial mouse-anti-c-myc antibody (A00704, ThermoFisher, 1:5000 dilution) as primary antibody and goat-anti-mouse-Fc polyclonal antibody mixture labelled with alkaline phosphatase (Jackson, UK, 1:5000 dilution) as secondary antibody. Subsequently, a nitroblue tetrazolium and 5-bromo-4-chloro-3-indolyl phosphate solution was used to develop protein-specific staining.

Mouse-anti-c-myc antibody 9E10 or peptides (Table 1) were immobilized on amine chips by EDC/NHS coupling (Table S3) for use in surface plasmon resonance (SPR) spectroscopy. ELP fusion proteins and 9E10 antibody samples were diluted in running buffer (10 mM HEPES, 3 mM EDTA, 150 mM sodium chloride, 0.05% (v/v) Tween-20, pH 7.4) and quantified by SPR spectroscopy on a Sierra SPR 2 device (Bruker (formerly Sierra Sensors), Hamburg; Germany) using antibody standards in the 0.25–10.00 mg L^−1^ range.

**Table 1:** Ligands used for the quantification of ELP fusion proteins by SPR assays

	Unit	Immobilized ligand			
		Peptide 1	Peptide 2	Peptide 3	mAb 9E10
Specificity	[–]	Antigen (part of c-myc) recognized by antibody 9E10			c-myc
Sequence^a^	[–]	*NH2*-GGEQKLISEEDLN-*COOH*	*CH3CO*-RRGEQKLISEFELN-*CONH2*	*NH2*-KRGEQRLISEFELN-*COOH*	Not shown
Molecular mass	[kDa]	1.43	1.72	1.72	148.92
Sequence modifications	[–]	none	Acetylated N-terminus and C-terminal amide and additional arginine at the N-terminus^b^	Additional lysine and arginine residue at the N-terminus^c^	none
Ligand immobilization	[RU]	20	250	380	1500
Ligand density	[g m^−2^]	0.02 × 10^–3^	0.25 × 10^–3^	0.38 × 10^–3^	1.50 × 10^–3^
Ligand density	[M m^−2^]	14 × 10^–9^	145 × 10^–9^	221 × 10^–9^	10 × 10^–9^
Maximal 9E10 binding signal	[RU]	9	115	700^d^	n.a
Regeneration stability	[# of cycles]	n.d	50	> 100	20

The peptides contained the fraction of c-myc representing the epitope of mAb 9E10 and were used as ligands during indirect SPR assays. Peptide sequences were selected based on a previous report (Krauss et al. 2008)

^a^The N-terminal and C-terminal functional groups of the peptides are shown in italics and represent the corresponding atoms whereas the rest of the sequence (regular font) represents one-letter amino acid code. ^b^these modifications of the peptide allowed a coupling in both orientations (either C-terminus or N-terminus in proximity to the chip surface) so minimize potential steric hindrance during antibody binding. ^c^ this modification increases the number of free amine groups available for coupling. The core epitope recognized by antibody 9E10 is underlined. ^d^No saturation was observed during immobilization

For SPR competition measurements, 1:40 (v/v) diluted process samples were mixed 1:1 (v/v) with 5.0 mg·L^−1^ (33.6 nM) 9E10 antibody or 2.5 mg L^−1^ (16.8 nM) anti-c-myc antibody (A00704, ThermoFisher). Standards of 100 × ELP were prepared as a serial dilution in the 8–2000 µg·L^−1^ (0.2–43.9 nM) range. Standards and process samples were then incubated for 16 h before injection. The signal of standard samples was taken for competition curve fitting using a one site competition function (Eq. 1) in Origin 8.1 (OriginLab Corporation, Northampton, USA), where y is the SPR signal in response or resonance units (RU), x is the molar ELP concentration, A_1_ and A_2_ are the upper and lower boundary of the sigmoidal function respectively, log x_0_ is molar ELP concentration at the inflection point of the function.

1 $$y = A_2 + \frac{A_1 - A_2 }{{1 + 10^{\left( {Log_{10} x - Log_{10} x_0 } \right)} }}.$$

## Results and discussion

### ELP fusion proteins can be quantified by an SPR competition assay

We first set out to establish a reliable quantitation assay for ELP fusion proteins based on a myc-tag present in all constructs (Fig. 2A). This was important because common detection methods such as densitometric analysis of Coomassie-stained polyacrylamide gels have proven insensitive to ELP-containing proteins, probably because the latter contain < 2% basic amino acids, which are necessary for binding the Coomassie dye (Hassouneh et al. 2010). Initially, we immobilized mouse-anti-c-myc antibody 9E10 (Krauss et al. 2008; Hilpert et al. 2001) on an amine sensor chip with dextran coating (the latter can increase chip capacity) for a direct quantification assay (Fig. S1A) achieving up to 1500 RU (Fig. S1B) after coupling, which was equivalent to ~ 1.5 mg m^−2^ of mAb and good compared to previous reports (Schasfoort and Schasfoort 2017; Murphy et al. 2017; Opdensteinen et al. 2023). Using repeated injections of the same clarified plant extract sample containing VSO1ELP in a 1:20 dilution in SPR running buffer, we found that the RU signal declined in the course of 150 runs from 790 to 82 RU (Fig. S1C). Because the baseline signal was stable, we excluded leaching of mAb from the chip surface as a reason for this reduction. Instead, we assumed that the required regeneration conditions (30 mM hydrogen chloride, Table S3) were too harsh for mAb 9E10 causing its denaturation (Lazar et al. 2010) and thus signal reduction. We therefore deemed this direct assay inadequate for ELP quantitation.

Next, we tested an indirect SPR assay where a defined amount of anti-c-myc antibody was added to ELP fusion protein samples and the resulting antibody binding to a chip surface covered with peptides containing the c-myc epitope was measured (Fig. 2B). Because of their small size, these peptides did not have a distinct three-dimensional structure so denaturation was not an issue during the regeneration of the sensor chip surface. However, the small size may also limit the number and steric accessibility of functional groups for coupling to the chip surface. We therefore tested three peptide variants (Table 1) that we developed based on previous recommendations (Krauss et al. 2008). The highest coupling response and signal stability over repeated sample injections was observed for peptide 3 (Fig. S1D), which then we used for all subsequent quantifications.

We confirmed that the competition assay provided quantitative results by establishing a high-quality standard curve (adj. R^2^ > 0.99; Eq. 1; Fig. 2C) using defined mixtures of an 100xELP standard with antibody 9E10 as well as a commercially available anti-c-myc antibody A00704 as reference material. The inflection points of the models were ~ 5 × 10^–8^ M (9E10) and ~ 3 × 10^–8^ M (A00704) corresponding to ~ 250 and ~ 350 µg L^−1^ of 100xELP respectively, which marked the most reliable quantification region of the assay. Given TSP levels of ~ 12 g kg^−1^ in tobacco biomass (Opdensteinen et al. 2018) and fusion protein expression levels of up to 0.02–1.00% TSP in previous work (Phan et al. 2013), a 1:4 dilution during extraction and a 1:40 dilution during sample preparation, we expected product concentrations of 15–750 µg L^−1^ during measurement and concluded that this matched well with the quantitation range of the assay.

### A modified clarification process is necessary to avoid product losses

We then individually expressed the five fusion proteins VSO1ELP, MaSp1ELP, MaSp2ELP, FlagELP and 100xELP in transgenic tobacco plants and observed product levels of ~ 0.02 (MaSp2ELP) to 0.60 (100xELP) g kg^−1^ biomass (VSO1ELP ~ 0.40 g kg^−1^; MaSp1ELP ~ 0.45 g kg^−1^; FlagELP ~ 0.30 g kg^−1^), which was slightly higher compared to previous reports where ELP fusion proteins accumulated up to 0.01–0.12 g kg^−1^ (Phan and Conrad 2011; Phan et al. 2013). We extracted the products with a buffer containing salt in a concentration required for the subsequent mITP (1.50 to 3.00 M sodium chloride) to streamline the process. A sequence of bag and depth filtration was used for clarification as described before (Buyel and Fischer 2014b). Interestingly, the recovery of ELP fusion proteins was low under these conditions, for example < 5% for VSO1ELP (data not shown). We first speculated that even at ~ 22 °C the high salt concentrations may have caused some degree of ELP fusion protein aggregation as reported before (Christensen et al. 2013). This could result in a retention of the product on the filters, especially as the latter had nominal pore diameters of < 1.0 µm, which is about the size of ELP aggregates (Hassouneh et al. 2010, 2012; Dreher et al. 2008).

**Fig. 3:**
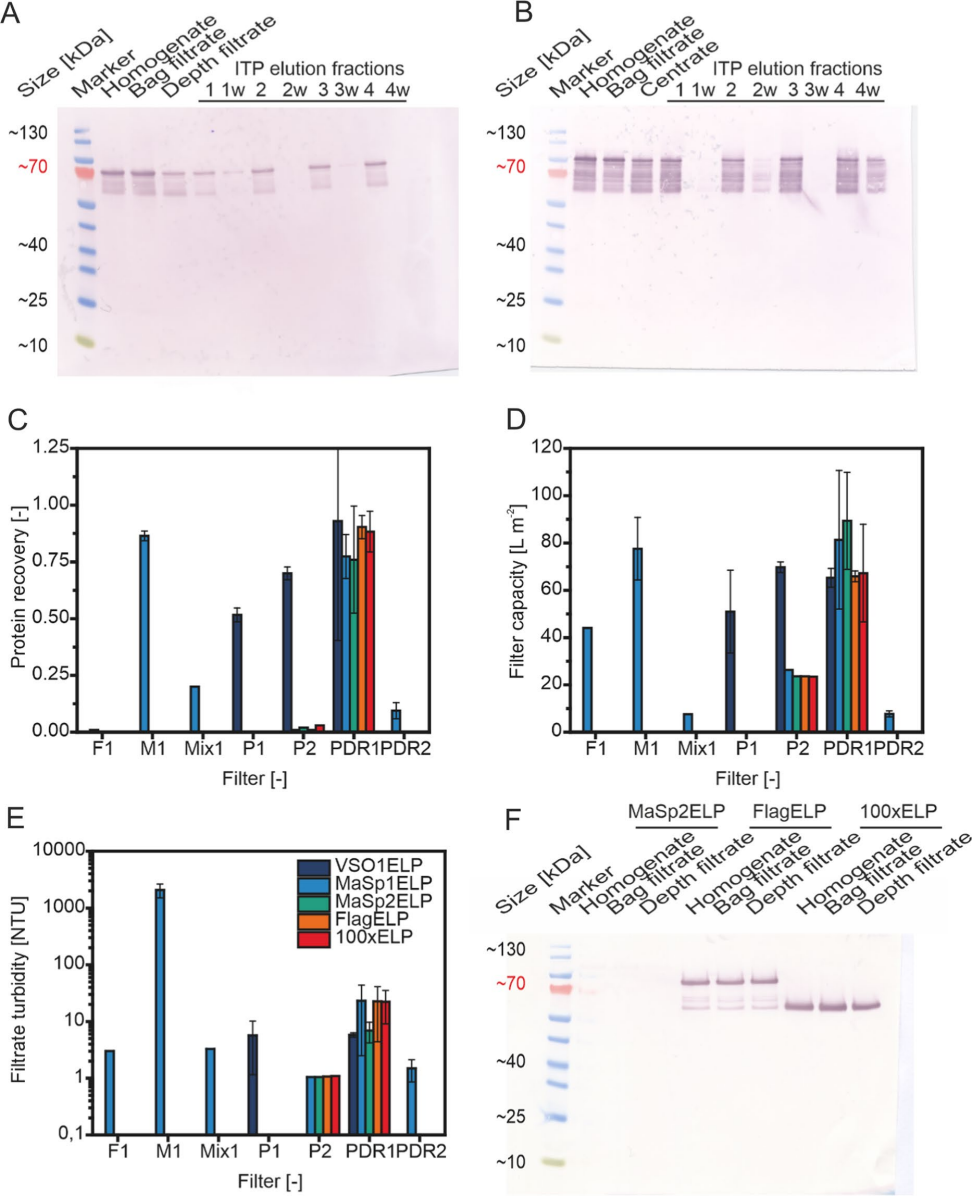
Screening of depth filters and ELP fusion protein recovery during clarification. **A** Western blot of process samples using depth filter P1 and anti-c-myc for detection of VSO1ELP. Elution fractions originated from 1&3—0.2 µm membrane pore size, 2.0 M sodium chloride during aggregation, 30 °C during wash; 2&4—1.2 µm membrane pore size, 2.4 M sodium chloride during aggregation, 45 °C during wash; primary elution was carried out using 15 mM sodium phosphate buffer pH 7.5 whereas de-ionized water (indicated by “w”) was used for a second elution step. **B** Western blot of process samples using centrifugation and anti-c-myc for detection of MaSp1ELP. Elution fraction conditions as in A. **C** ELP fusion protein recovery achieved with different depth filters (Table S1). **D** Filter capacity in dependence of filter layer combinations and ELP fusion protein. **E** Turbidity observed after clarifying ELP fusion protein containing extract with different depth filters. Error bars in **C**–**E** indicate the standard deviation (n ≥ 3). **F** Western blot of process samples using depth filter PDR1 and anti-c-myc for detection of ELP fusion proteins. *ELP* elastin-like polypeptide, *ITP* inverse transition purification. Error bars indicate the standard deviation of replicate runs with n ≥ 2

Therefore, we shifted the salt addition to after depth filtration rather than before extraction for all subsequent experiments. This restored the recovery of VSO1ELP to 56 ± 9% (n = 9; Fig. 3A), whereas the recovery remained < 5% for all other ELP fusion proteins unless depth filtration was replaced by centrifugation (Fig. S2), as was the case for MaSp1ELP (Fig. 3B). It seemed implausible to us that these proteins would form aggregates due to the base salinity of the extraction buffer (~ 20 mS cm^−1^) at 22 °C. Instead, unspecific binding of proteins to depth filters containing diatomaceous earth has been reported before (Knödler et al. 2023; Opdensteinen et al. 2021), and we then assumed that electrostatic interactions between the proteins and the charged components of the depth filter caused the product losses as previously observed for other molecules (Menzel et al. 2018). We therefore tested a set of depth filters that contained less diatomaceous earth (Fig. 3C–E, Table S1), because this component can absorb proteins (Yigzaw et al. 2006; Buyel et al. 2015). Filter PDR1 performed best, combining a high filter capacity (> 60 L m^−2^), low turbidity (< 25 NTU) and high product recovery (> 0.75) (Fig. 3F). This filter was then used for all subsequent experiments also because it was easier to scale up compared to centrifugation which achieved a similar product recovery.

### Different membranes and aggregation conditions can be used for fusion protein purification

**Fig. 4:**
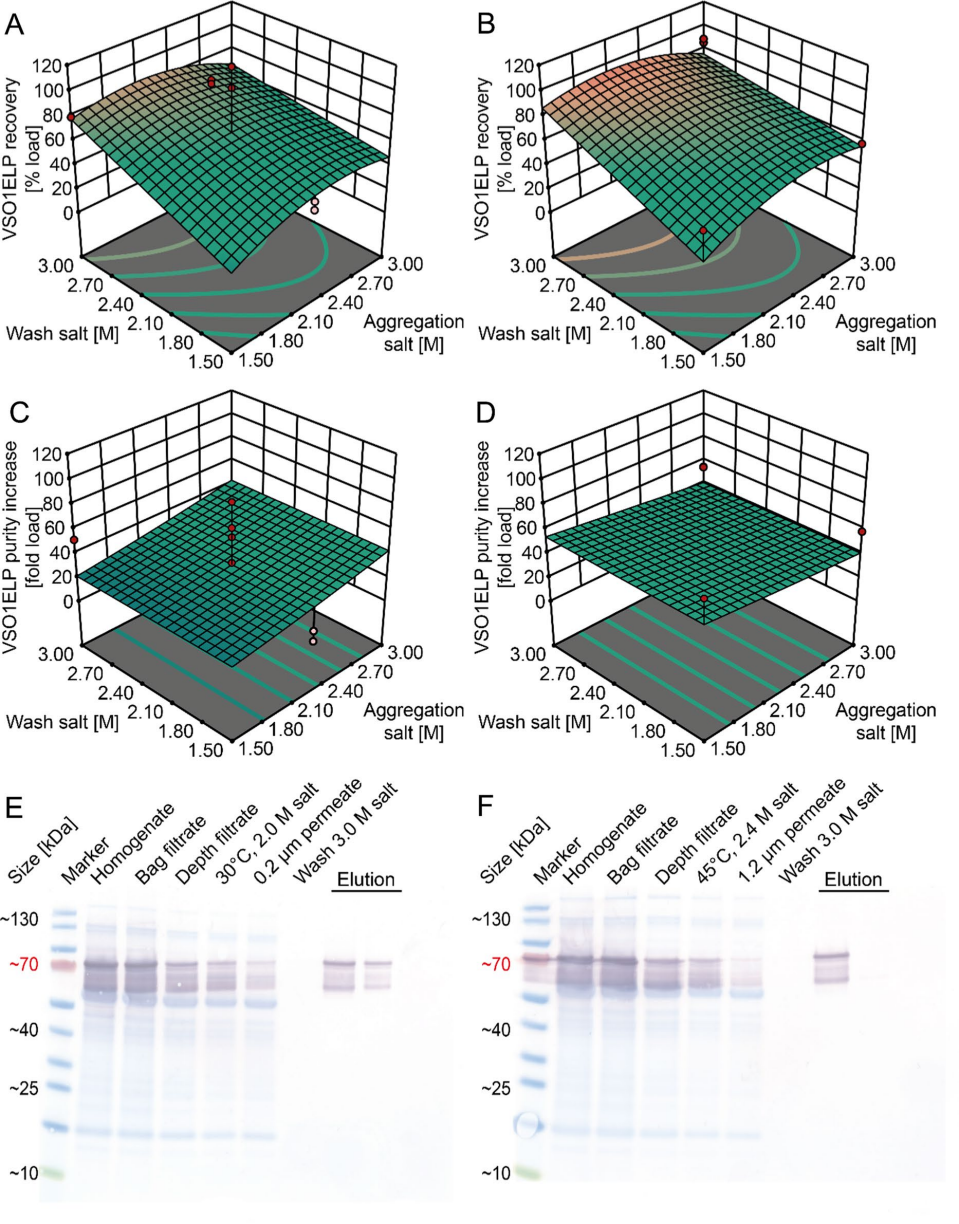
Screening for mITP conditions ensuring a high recovery and purity of ELP fusion proteins using VSO1ELP as a model protein. **A** Response surface model for VSO1ELP recovery in dependence of sodium chloride concentration during aggregation and wash using a 0.2-µm membrane for aggregate retention. **B** Same model as in A but using a 1.2-µm membrane. **C** Response surface model for VSO1ELP purity increase as a multiple of the starting purity in dependence of sodium chloride concentration during aggregation and wash using a 0.2-µm membrane for aggregate retention. **D** Same model as in C but using a 1.2-µm membrane. The aggregation temperature did not have a significant effect in the 30–45 °C range and was set to 37.5 °C in panels **A**–**D**. Dots indicate actual measurements. **E** Overlay of Coomassie-stained LDS-PAA gel and corresponding western blot using anti-c-myc antibody for VSO1ELP detection in process samples for the verification of the process optimum using a 0.2-µm membrane. **F** Same setup as in E but with samples from runs confirming the optimum for a 1.2-µm membrane

We used VSO1ELP for an initial statistical screening experiment to identify suitable conditions for mITP, achieving high purity and recovery. Extract clarified by bag filtration and depth filtration with PDR1 was used to test different mITP conditions (Table S2). The resulting model for product recovery was of fair quality given the complex sample preparation and small scale of the experiments (adj. R^2^ 0.56; Table S4). The model indicated that the salt concentration during wash was the dominating factor for VSO1ELP recovery and that aggregation salt concentration and membrane pore sizes had a smaller effect. The aggregation temperature did not have a significant contribution in the investigated range (30–45 °C). High VSO1ELP recoveries were identified for two different factor combinations, both performing aggregation and wash at 2.0 M and 3.0 M respectively, but using either a 1.20 µm or 0.20 µm membrane (Table S2, Fig. 4A and B).

We also analyzed VSO1ELP purity in mITP elution fractions and found that hardly any protein was detected on Coomassie-stained LDS-PAA gels (Fig. 4C and D) but that silver-staining revealed a dominant band of the size expected for VSO1ELP (~ 70 kDa) as well as a smaller band at ~ 55 kDa. Because the latter band was also detected by western blotting and had a similar size as the 100xELP protein which did not contain a spider silk fusion part, we assume that it corresponded to a product-related degradation (Fig. 3E and F). Combining the detection limit of Coomassie brilliant blue-based protein staining of ~ 0.01 g L^−1^ (Opdensteinen et al. 2018) with the VSO1ELP quantification by SPR, we estimated the minimal VSO1ELP purity to be > 90%. This was in good agreement with previous reports using mITC that achieved purities of up to 97% (Phan et al. 2013). Interestingly, no significant model was obtained for the product purity. We concluded that the mean purity was the best estimator and that model factors did not have a relevant influence on VSO1ELP purity in the investigated ranges.

The two optimal conditions in terms of VSO1ELP recovery were verified in independent runs and yielded recoveries of 114 ± 28% (1.2-µm membrane) and 86 ± 19% (0.2-µm membrane) respectively (n = 3). The high recoveries were in good agreement with previous reports for mITC where up to 90% were reported (Phan and Conrad 2011; Floss et al. 2009).

### Effective mITP conditions can be identified for individual ELP fusion proteins

We then conducted an additional design of experiments to adapt the two optimal conditions to other fusion proteins MaSp1ELP, MaSp2ELP, FlagELP and 100xELP. Because 1.2-µm membranes were not available from the manufacturer of the ultrafiltration/diafiltration (UF/DF) device for a subsequent scale-up, we limited the investigation to the aggregation temperature and salt concentration but kept the wash salt concentration at 3.0 M, the optimal level for all membranes. The recovery of MaSp1ELP was only affected by the aggregation salt concentration (adj. R^2^ = 0.89) with an optimum at ~ 2.7 M sodium chloride (> 90% recovery), whereas there were no significant effects on the recovery of FlagELP or 100xELP which were ~ 95% and 70% respectively. A model for MaSp2 was not established because the expression level and concentration after depth filtration of the recombinant protein were too low to allow a quantification.

## Conclusions

Here we have developed an SPR-based quantitation method for ELP fusion proteins, which are difficult to detect using conventional staining methods. This will accelerate future process development because the performance of individual unit operations can be rapidly assessed, e.g. in terms of purity and recovery.

We have also established a fast, membrane-based purification method for ELP fusion proteins that can simplify manufacturing, e.g. for future technical applications of spider silk proteins. Specifically, a repeated cycling of ELP fusion proteins between aggregated and dissolved state can be avoided during purification as impurities are flushed out much like in a regular UF/DF operation. Because the method uses readily available membranes of 0.2 µm pore diameter, an implementation into production processes seems straight forward. Once 1.2 µm membranes become commercially available for UF/DF devices, the throughput of the system may be increased, e.g. due to a reduction in membrane fouling and concentration polarization (Kim 2007). Quantifying such unwanted side effects along with typical loadings (i.e. grams of aggregated ELP fusion protein per square meter of membrane area) should be the focus of subsequent scale-up experiments, for example using tangential flow filtration devices. In this context, the impact of pH during wash and aggregation of ELP fusion proteins can be assessed too, but may have little effect due the uncharged amino acids constituting the ELP tag. Additionally, implementing the removal of the ELP (and tag) fusion parts in the process, for example through (self-)cleavage (Lingg et al. 2022; Li 2011), will be necessary in the future.

### Supplementary Information

Below is the link to the electronic supplementary material.

Supplementary file1 (DOCX 1580 kb)

## Data Availability

Data can be made available upon request to the corresponding author.

## Abbreviations

EDC: 1-Ethyl-3-(3-dimethylaminopropyl)carbodiimide
ELP: Elastin-like polypeptide
HCP: Host cell protein
ITC: Inverse transition cycling
ITP: Inverse transition purification
NHS: N-hydroxysuccinimide
RU: Resonance unit
SPR: Surface plasmon resonance
TSP: Total soluble protein
UF/DF: Ultrafiltration/diafiltration
