# Supplementary material for: Membrane-based inverse-transition purification facilitates a rapid isolation of various spider-silk elastin-like polypeptide fusion proteins from extracts of transgenic tobacco
Source: Transgenic Res. 2024 Apr 4;33(1-2):21–33. doi: 10.1007/s11248-024-00375-z (PMC11021290; doi:10.1007/s11248-024-00375-z)
Supplement: Supplementary file 1 — Supplementary file1 (DOCX 1580 kb) [file 11248_2024_375_MOESM1_ESM.docx]

**Supplementary data**

Table S1. Depth filters tested for the clarification of ELP fusion protein containing plant extract.

| Filter | Unit | F1 | M1 | Mix1 | P1 | P2 | PDR1 | PDR2 |
| --- | --- | --- | --- | --- | --- | --- | --- | --- |
| First layer | [-] | CH71HP | CE45 | CE40 | K200P | K200P | K700P | Bio20 |
| Nominal pore size | [µm] | 1.5-3.0 | 0.9-2.0 | 1.2-3.0 | 3.0-6.0 | 3.0-6.0 | 6.0-15.0 | 0.4-1.0 |
| DE content | [-] | High | None | None | Medium | Medium | Low | None |
| Manufacturer | [-] | Filtrox | Merck-Millipore | Merck-Millipore | Pall | Pall | Pall | Pall |
| Second layer | [-] | CHST110 | CE50 | Bio20 | KS50P | K100P | V100P | Bio10 |
| Nominal pore size | [µm] | 0.5-0.8 | 0.6-1.2 | 0.4-1.0 | 0.5-1.5 | 1.0-3.0 | 2.0-4.0 | 0.2-0.4 |
| DE content | [-] | High | None | None | High | Medium | Low | None |
| Manufacturer | [-] | Filtrox | Merck-Millipore | Pall | Pall | Pall | Pall | Pall |

Table S2. Factors and their ranges tested during mITP optimization.

|  |  | Level | | | | Optima | |
| --- | --- | --- | --- | --- | --- | --- | --- |
| Factor | Unit | 1 | 2 | 3 | 4 | 1 | 2 |
| Aggregation temperature | [°C] | 30.0 | 37.5 | 45.0 | n.a. | in range | |
| Aggregation salt concentration | [M] | 1.50 | 2.25 | 3.00 | n.a. | 2.00 | |
| Wash salt concentration | [M] | 1.50 | 2.25 | 3.00 | n.a. | 3.00 | |
| Membrane pore size | [m] | 0.20×10^-6^ | 0.45×10^-6^ | 0.80×10^-6^ | 1.20×10^-6^ | 0.20×10^-6^ | 1.20×10^-6^ |

Table S3. Conditions applied for ligand coupling to SPR amine flat sensor (from Bruker, the chip surface is equivalent to that of other manufacturers, e.g. Sensor Chip CM5 from Cytiva) chip surfaces and subsequent sample analysis.

|  |  |  | Ligand | | | |
| --- | --- | --- | --- | --- | --- | --- |
| Step | Parameter | Unit | Peptide 1 | Peptide 2 | Peptide 3 | 9E10 |
| EDC/NHS activation | Ligand concentration | [mg L^-1^] | 200 | 200 | 200 | 50 |
|  | Sodium acetate buffer pH | [-] | 5,5 | 5 | 5,5 | 4,5 |
|  | Contact time | [min] | 12 | 12 | 12 | 12 |
|  | Flowrate | [µL min^-1^] | 10 | 10 | 10 | 10 |
|  | Reagent concentration | [M] | 0.05/0.20 | 0.05/0.20 | 0.05/0.20 | 0.05/0.20 |
| Ligand contact | Contact time | [min] | 12 | 12 | 12 | 7 |
|  | Flowrate | [µL min^-1^] | 10 | 10 | 10 | 15 |
| Ethanolamine deactivation | Contact time | [min] | 7 | 7 | 7 | 7 |
|  | Flowrate | [µL min^-1^] | 5 | 5 | 5 | 5 |
|  | Reagent concentration | [M] | 1 | 1 | 1 | 1 |
| Sample injection | Contact time | [min] | 3 | 3 | 3 | 3 |
|  | Flowrate | [µL min^-1^] | 30 | 30 | 30 | 30 |
| Surface regeneration with hydrogen chloride | Contact time | [min] | 1 | 1 | 1 | 1 |
|  | Flowrate | [µL min^-1^] | 30 | 30 | 30 | 30 |
|  | Reagent concentration | [M] | 0.03 | 0.03 | 0.03 | 0.03 |

Table S4. Descriptive model for the prediction of VSO1ELP recovery during mITP under different conditions.

| Factor | F-value | p-value |
| --- | --- | --- |
| Model | 12.436 | 0.000 |
| Aggregation salt concentration (B) | 0.941 | 0.336 |
| Wash salt concentration (C) | 33.494 | 0.000 |
| Membrane pore size (D) | 7.371 | 0.009 |
| BC | 5.700 | 0.020 |
| B² | 6.383 | 0.014 |
| D² | 3.638 | 0.062 |
| D³ | 5.466 | 0.023 |
| Quality indicator | p-value | |
| Lack of Fit | 0.002 | |
| R² | 0.604 | |
| Adjusted R² | 0.556 | |
| Predicted R² | 0.417 | |


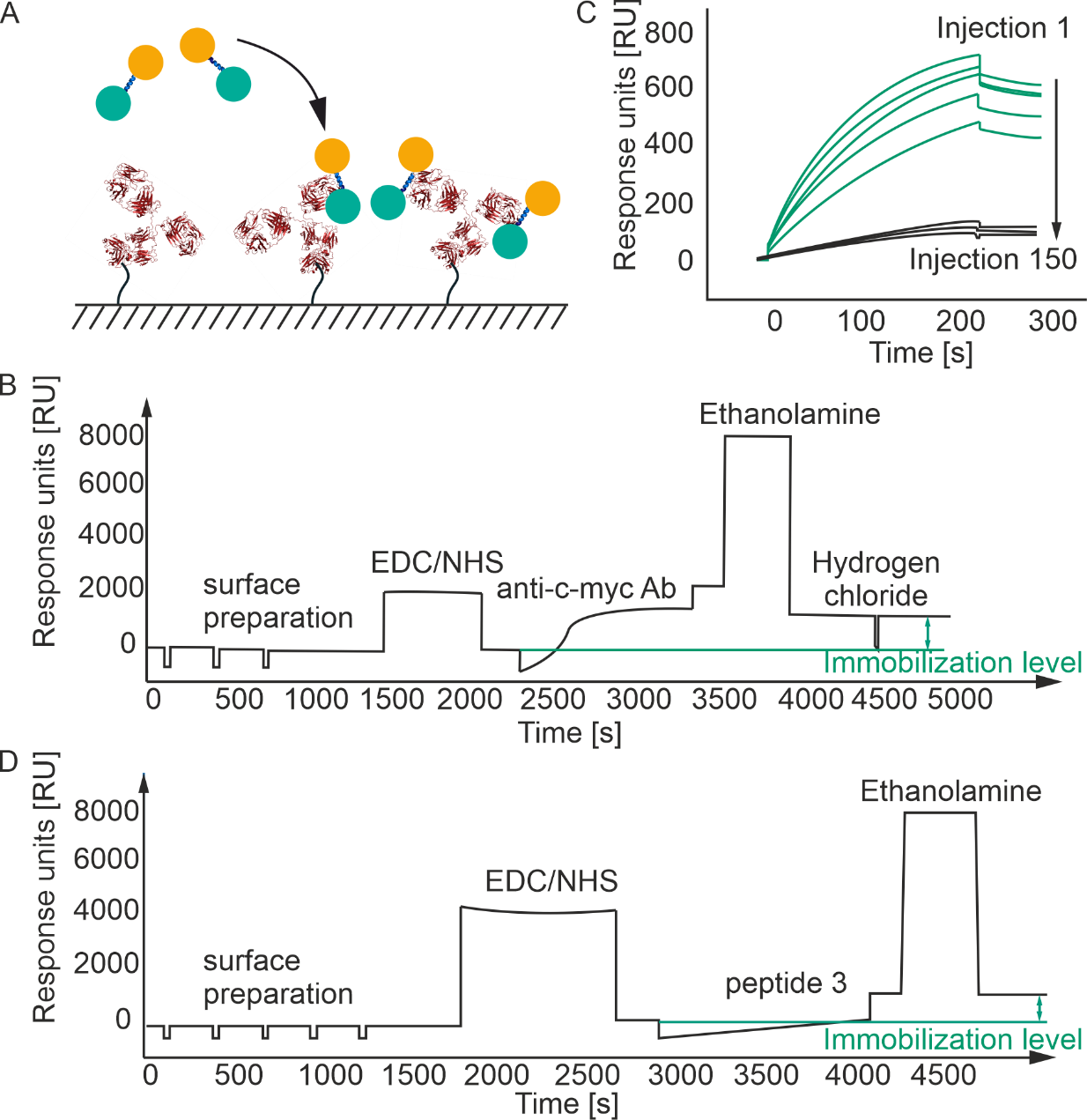


Figure S1. Development of a surface plasmon resonance (SPR) spectroscopy assay for ELP fusion protein quantitation. A. Schematic representation of a direct binding assay. Monoclonal antibody 9E10 (red) is immobilized on an the SPR chip surface by EDC/NHS coupling and can capture spider silk-ELP fusion proteins (green and orange domains respectively) that contain a c-myc tag (blue). B. Trace of the resonance signal during 9E10 coupling to an SPR chip surface. The individual steps are labeled (see also **Table S3**). C. Stability of the resonance signal after 9E10 coupling in the course of repeated sample injections and regeneration cycles. D. Exemplary trace of the resonance signal during peptide coupling to an SPR chip surface.


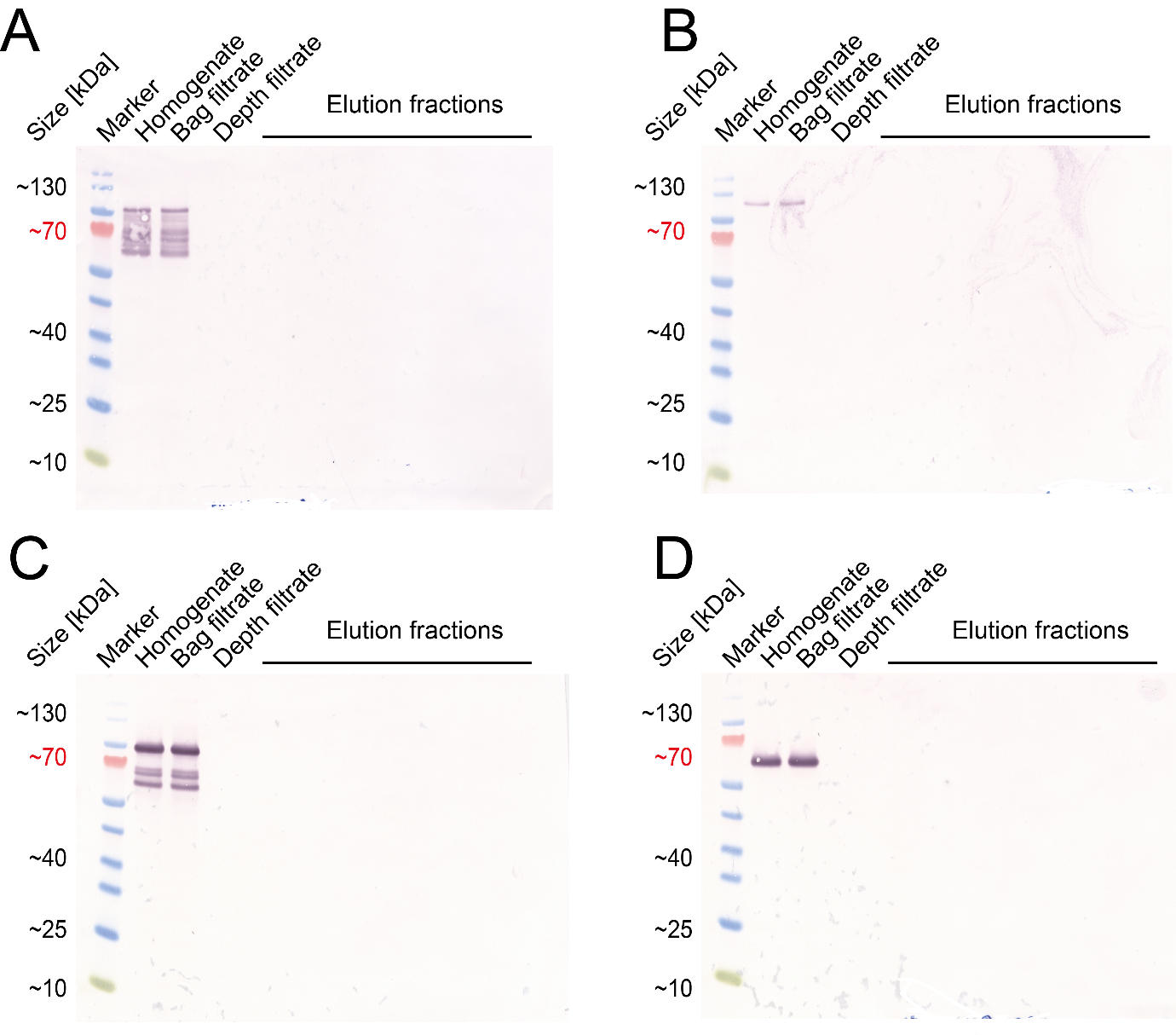


Figure S2. Recovery of ELP fusion proteins during clarification and purification using depth filter P1 (**Table S1**) that contains diatomaceous earth. Elution fractions are samples obtained from mITP after depth filtration. Mouse-anti-c-myc was used as primary antibody for MaSp1ELP (A), MaSp2ELP (B), FlagELP (C) and 100xELP (D).
